# Barriers to cardiac rehabilitation among adults with heart failure and nursing-led strategies to improve participation: a systematic review

**DOI:** 10.3389/fcvm.2026.1932972

**Published:** 2026-10-27

**Authors:** Hiba Ahmed, Anas Alsharawneh, Laila Ibraheem Jaber Salameh

**Affiliations:** 1 Hind Bint Maktoum College of Nursing and Midwifery, Mohammed Bin Rashid University of Medicine and Health Sciences, Dubai Health, Dubai, United Arab Emirates; 2 Department of Adult Health Nursing, Faculty of Nursing, The Hashemite University, Zarqa, Jordan; 3 Pulmonary Department, Rashid Hospital, Dubai Health, Dubai, United Arab Emirates

**Keywords:** heart failure, cardiac rehabilitation, cardiovascular nursing, systematic review, barriers, adherence, referral, health equity

## Abstract

**Background:**

Heart failure (HF) is a major cause of morbidity, reduced quality of life, and repeated hospital admissions worldwide. Cardiac rehabilitation (CR) is a guideline-recommended intervention that improves functional capacity, quality of life, and clinical outcomes in patients with HF. Despite its proven benefits, participation in CR remains consistently low, suggesting the presence of significant barriers at patient, provider, organizational, and health-system levels. Nurses play a central role throughout the heart failure care continuum and are well positioned to influence referral, enrolment, adherence, and long-term engagement in rehabilitation programmes.

**Aim:**

This systematic review aimed to identify barriers affecting participation in cardiac rehabilitation among adults with heart failure and to examine the role of nursing in improving referral, enrolment, attendance, adherence, and programme completion.

**Methods:**

A systematic review was conducted in accordance with the Preferred Reporting Items for Systematic Reviews and Meta-Analyses (PRISMA 2020) guidelines. Five electronic databases (PubMed, CINAHL, EMBASE, Scopus, and MEDLINE) were searched for peer-reviewed studies published between 2015 and 2025. Studies were eligible if they involved adults with heart failure, investigated barriers to cardiac rehabilitation participation, and included a nursing perspective, role, or intervention. Methodological quality was assessed using the Mixed Methods Appraisal Tool (MMAT 2018). Data were synthesized using narrative and thematic analysis.

**Results:**

From 1,509 identified records, 17 studies met the inclusion criteria. Four major themes emerged: patient-related barriers, systemic and structural barriers, socio-cultural and equity-related barriers, and nursing-related facilitators. Common barriers included health literacy limitations, symptom burden, exercise-related fear, comorbidities, fragmented referral pathways, limited-service availability, geographic isolation, transportation difficulties, digital accessibility challenges, language barriers, and socioeconomic disadvantages. Women, migrants, rural populations, and socially vulnerable groups were disproportionately affected. Facilitators included nurse-led care coordination, patient education, motivational support, structured follow-up, automatic referral systems, and home-based, hybrid, and digitally enabled rehabilitation models supported by ongoing clinical supervision.

**Conclusion:**

Participation in cardiac rehabilitation among patients with heart failure is influenced by complex and interconnected barriers that extend beyond individual motivation. Nurses play a critical role in addressing these challenges through education, care coordination, behavioral support, and advocacy. Strengthening nurse-led interventions and expanding flexible, equitable rehabilitation models may improve cardiac rehabilitation referral, engagement, and adherence among people living with heart failure.

## 1 Introduction

Cardiovascular disease continues to dominate global mortality statistics accounting for approximately 32% of all global deaths (1). Of all heart diseases, Heart failure (HF) is the most intricate expression of cardiovascular disease, affecting over 64 million people worldwide (2). Heart failure is a clinical condition characterised by structural or functional abnormalities of the heart which induces the inability of the heart to pump or fill effectively hence poor tissue perfusion and/or increased intracardiac pressures (2). The The global burden of heart failure continues to increase and has been owed mainly by the ageing of the population, the survival rates of the acute cardiac events, and the rising occurrences of the comorbidity like the diabetes and hypertension. Consequently, HF causes are dominant hospitalisation in older individuals, with increased rate of exacerbations as well as readmission, which puts indefinite pressure on already strained health systems. Outside the economic consequences, HF has had a severe impact on the quality of the lives of patients, and it has led to developing fatigue, breathlessness, functional incapacity, psychological distress and social isolation (3). The pathophysiology of HF occurs after myocardial injury or chronic haemodynamic stress resulting in ventricular remodelling, neurohormonal stimulation and deterioration of cardiac output. The clinical syndrome of heart failure is marked by key symptoms that significantly hinder functional independence, particularly dyspnoea (shortness of breath), ongoing fatigue, and fluid retention. These symptoms frequently culminate in exercise intolerance, a state where even everyday tasks become burdensome because the heart cannot satisfy the body's metabolic requirements (4, 5).

Sex and gender have also been identified as important determinants of cardiac rehabilitation participation, influencing referral patterns, access, and engagement, yet are frequently underreported or insufficiently explored within the heart failure rehabilitation literature (6, 7).

As these symptoms intensify, NYHA Functional Classification patients generally develop physical deconditioning and ‘fear-avoidance,’ limiting their mobility to avoid pain. This physical deterioration frequently coincides with notable psychological distress, such as anxiety and depression, which further diminishes the patient's quality of life. These clinical challenges are what cardiac rehabilitation seeks to tackle by enhancing peak oxygen consumption (VO_2_ peak) and improving the efficiency of peripheral muscles to regain functional ability (8).

Moreover, HF has an enormous economic cost of approximately $284.17 billion spent on healthcare each year in terms of hospitalisations, pharmacotherapy and long-term management (9). In the United Kingdom, HF causes a lot of burden to the National Health Service (NHS) which has been significantly adding tens of thousands of admissions to the country per annum and much use of resources. There are also similar trends of increase in Europe and Middle East with rapid epidemiological transition and lifestyle shifts that are rapidly accelerating cardiovascular risk patterns. In the United Kingdom, heart failure poses a substantial burden on the National Health Service, accounting for tens of thousands of hospital admissions annually and extensive resource utilization, with similar trends observed across Europe and the Middle East (10, 11).

Cardiac rehabilitation (CR) has broadly been generated in reaction to the growing load of HF to complement secondary prevention and management of chronic illnesses (10). CR is a multidisciplinary programme involving supervised training of the exercises, education to the patients, cardiovascular risk reduction, lifestyle modification and psychosocial support. Effective evidence is shown that participation of CR in HF populations leads to an increase in functional capacity, quality of life, reduction in hospital readmissions, and all-cause, and cardiovascular mortality reduction by about 20-30 percent (12). Patients that are having CR also exhibit better self-management behaviours and less symptom burden. World health organisation, European society of cardiology and American heart association guidelines are very much in support of CR to qualified HF patients (13). But despite such established advantages and guideline recommendations, CR remains substantially underutilized all over the world, with the rate of participation being lowest in HF populations. Several overlapping barriers such as patient level, organisational, and socio-cultural cause this consistent evidence-practice gap. Given their role as the primary organisers of chronic disease management and educators of patients, nurses have a unique opportunity to impact referral and engagement and adherence to CR programmes (14). Since the world is rapidly increasing in ageing population, and because chronic cardiovascular disease is increasingly becoming widespread, the study of barriers to CR participation in HF, as well as how these barriers are timely and of utmost importance in enhancing patient satisfaction and transforming nursing practice. Nurses play a key role in coordinating care during the vital shift from acute heart failure treatment to ongoing community rehabilitation. Cardiovascular nurses, as the primary point of patient interaction, are specially equipped to recognize individual obstacles promptly through clinical evaluation and symptom tracking (5). In addition to medical management, the nursing role includes health education, motivational interviewing, and psychosocial support, which are vital for enabling patients to address ‘fear-avoidance' behaviours linked to dyspnoea and fatigue. As advocates and educators for patients, nurses can directly impact referral rates and compliance, ensuring that the diverse needs of heart failure patients are addressed within a multidisciplinary team (15).

Despite extensive research on barriers to participation in cardiac rehabilitation (CR), evidence remains fragmented across different aspects of heart failure (HF) care. Existing studies have explored patient-related, organisational, and socio-cultural barriers, while others have focused on referral pathways, programme accessibility, or rehabilitation outcomes in broader cardiac populations (16). However, HF patients experience unique challenges such as symptom instability, frailty, multiple comorbidities, and frequent hospital readmissions, all of which may influence engagement with CR differently from other cardiac conditions (17). In addition, studies examining the role of nurses in CR participation are dispersed across various healthcare contexts, including discharge planning, patient education, motivation, and long-term chronic disease management. Bringing together this evidence is important to provide a clearer understanding of the barriers affecting CR participation specifically among adults with HF and to identify how nursing practice can support referral, enrolment, and adherence to rehabilitation programmes. This review therefore aims to synthesise existing evidence to address these interconnected aspects comprehensively

The choice of the nursing role as a central element for this review stems from the profession's key role in the heart failure care continuum. Nurses frequently serve as the main organizers of discharge planning and transition management; they successfully ‘unlock the door' to rehabilitation by being the first to recognize qualified patients and start the referral process. Nurses, who engage the longest with patients during acute exacerbations, are in an excellent position to recognize and address barriers like fear-avoidance or limited health literacy prior to the patient's discharge from the hospital. Consequently, a nursing-focused examination of CR obstacles is not just an academic task but an essential clinical requirement to enhance the adoption of secondary prevention in heart failure treatment (18, 19). The aim of this study is to critically analyse the impediments affecting attendance to Cardiac rehabilitation in adults diagnosed with Heart failure, and to investigate how the nursing profession can influence referral, enrolment and compliance with cardiac rehabilitation programmes.

## 2 Methodology

### 2.1 Protocol and registration

The review protocol was prospectively registered with PROSPERO (registration ID: CRD420261340335). The review was conducted and reported in accordance with the Preferred Reporting Items for Systematic Reviews and Meta-Analyses (PRISMA 2020) guidelines The completed PRISMA 2020 Checklist is provided in Supplementary File S1

### 2.2 Eligibility criteria

The population, interest, and outcome (PIO) framework was used to develop the eligibility criteria for this systematic review. The PIO framework is particularly suitable for systematic reviews that include qualitative, quantitative, and mixed-methods evidence and facilitates the identification of studies exploring experiences, barriers, and healthcare practices. The framework comprised

Population (P): Adult patients (≥18 years) diagnosed with heart failure and considered eligible for participation in cardiac rehabilitation programs.
Interest (I): Barriers to cardiac rehabilitation participation and nursing-related factors, including nurse-led care, nursing perspectives, nursing support, educational interventions, care coordination, and nursing actions that influence referral, enrolment, attendance, or adherence to cardiac rehabilitation.
Outcome (O): Identification of barriers affecting participation in cardiac rehabilitation, including factors related to referral, attendance, adherence, education, motivation, accessibility, and patient engagement.

#### Inclusion criteria

Studies were eligible for inclusion if they:

Were peer-reviewed and published between 2015 and 2025.
Were published in English
Included adult participants (≥18 years) with heart failure
Explicitly examined barriers to participation, access, or adherence in cardiac rehabilitation
Reported a nursing role, nursing perspective, nursing support strategy, or nurse-led intervention
Used qualitative, quantitative, or mixed-methods designs

#### Exclusion criteria

Studies were excluded if they met any of the following criteria:
Focused exclusively on cardiac conditions other than heart failure
Were systematic reviews, editorials, opinion pieces, conference abstracts, or case reports
Lacked full-text availability

### 2.3 Information sources

The literature search conducted using five electronic databases to ensure a comprehensive and systematic way to cover a wide range of pertinent nursing and health research. PubMed and MEDLINE were chosen due to its large indexing of biomedical and clinical studies regarding cardiovascular disease and rehabilitation. CINAHL was incorporated because of its high emphasis on nursing and other related health literatures, which makes it especially advantageous in researching the roles, as well as the views of nursing (20). Scopus was employed to not only get a substantial sample of interdisciplinary research but also to have the ability to access international research within healthcare systems. Strengthening coverage of high-quality peer-reviewed medical and nursing studies was also done in MEDLINE. Together, these databases provided comprehensive coverage of the literature relevant to cardiac rehabilitation and heart failure.

Grey literature was not systematically searched. The review focused on peer-reviewed studies indexed in major bibliographic databases to ensure methodological quality and reproducibility of findings. The literature review was restrictive to the studies published after 2015–2025 to represent the changing models of cardiac rehabilitation delivery, capture the effects of COVID-19 pandemic impacts on access to rehabilitation, and suit the modern nursing practice and policy settings.

### 2.4 Search strategy

In this systematic review, the search strategy involved the application of both Medical Subject Headings (MeSH) and free-text keywords to ensure comprehensive retrieval of relevant studies. MeSH terms facilitated the standardized indexing of concepts in databases such as PubMed and MEDLINE, while free-text keywords enabled the identification of articles that may not have been indexed under specific MeSH terms. The primary search terms included *Heart Failure*, *Cardiac Rehabilitation*, *Nursing*, *Barriers*, *Participation*, and *Adherence*, representing the key components of the research question. MeSH and free-text terms were combined using Boolean operators (AND/OR) to maximize search sensitivity and specificity (21). Searches were limited to English-language peer-reviewed studies published between 2015 and 2025. Studies were restricted to English-language publications due to feasibility constraints and to ensure accurate interpretation of qualitative findings during thematic synthesis. Ten full-text articles published in non-English languages were identified and excluded at the eligibility stage, as reflected in the PRISMA flow diagram. Although abstracts suggested potential relevance, full methodological details required for quality appraisal and data extraction were not consistently accessible. This limitation is acknowledged, particularly given the relative scarcity of evidence from low- and middle-income countries.

A representative search query applied in PubMed was as follows:

[“Heart Failure"[MeSH] OR “Heart Failure"[Title/Abstract]]
AND
[“Cardiac Rehabilitation"[MeSH] OR “Cardiac Rehabilitation"[Title/Abstract]]
AND
[“Nurses"[MeSH] OR “Nursing"[Title/Abstract]]
AND
[“Barriers"[Title/Abstract] OR “Participation"[Title/Abstract]
R “Adherence"[Title/Abstract]]

The complete search strategies used for all databases are provided in Supplementary Table S1.

### 2.5 Study selection

Study selection followed a two-stage screening process in accordance with the PRISMA 2020 framework. Titles and abstracts retrieved from PubMed, CINAHL, EMBASE, Scopus, and MEDLINE were initially screened against the predefined inclusion and exclusion criteria. Articles considered potentially eligible were subsequently subjected to full-text review to determine final inclusion.

To enhance methodological rigor and reduce selection bias, two independent reviewers conducted the screening process. Any disagreements were resolved through discussion and, where necessary, consultation with a third reviewer. Studies were excluded if they focused on pediatric populations, lacked a nursing role or perspective, or did not directly examine barriers to cardiac rehabilitation among patients with heart failure. The study selection process is presented in the PRISMA flow diagram (Figure 1).

**Figure 1:**
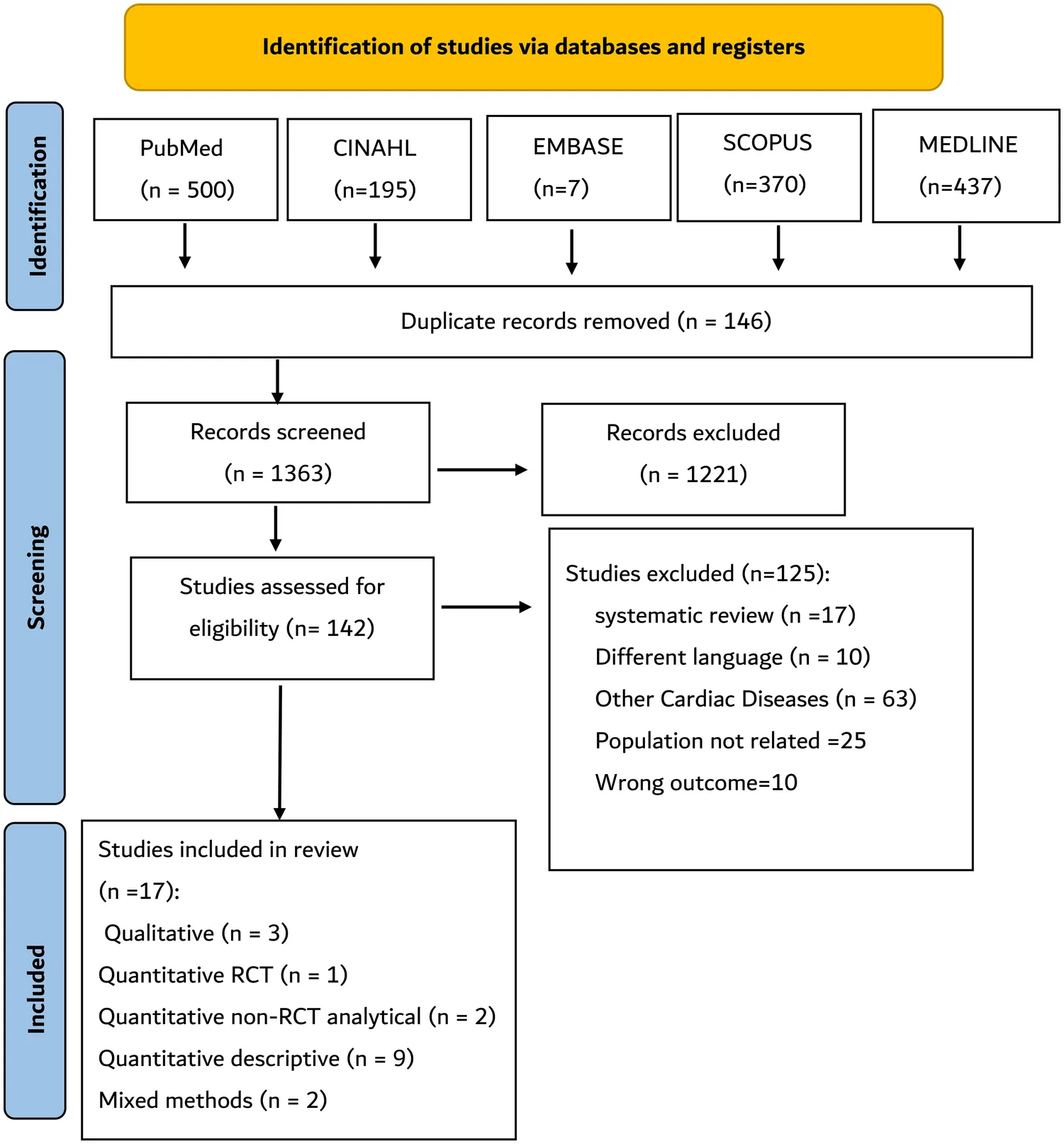
PRISMA flowchart depicting the study selection process according to inclusion and exclusion criteria.

### 2.6 Data extraction

A standardized data extraction form was developed to ensure consistency and transparency in the collection of relevant information from the included studies (Büchter et al., 2020). Microsoft Excel was used to organize, compare, and manage extracted data across all studies. Extracted variables included author(s), year of publication, country, study design, sample size and population characteristics, study objectives, identified barriers to cardiac rehabilitation, nursing roles or interventions, key findings, and study limitations. To optimize the extraction process, the data extraction form was piloted using two studies prior to full data extraction. Minor refinements were made to improve clarity and ensure that all relevant variables were captured. Data extraction was completed by the primary reviewer and independently verified by a second reviewer to ensure accuracy, reliability, and completeness. The selected variables were directly aligned with the review objectives and research questions concerning barriers to cardiac rehabilitation participation and the role of nursing in addressing these barriers.

### 2.7 Quality assessment

The methodological quality of the included studies was assessed using the Mixed Methods Appraisal Tool (MMAT) 2018 (22). The MMAT is widely used in systematic reviews incorporating qualitative, quantitative, and mixed-methods studies because it enables the evaluation of multiple study designs within a single framework. The tool assesses study quality using five design-specific methodological criteria, including the appropriateness of the research design, sampling strategy, data collection procedures, risk of bias, and adequacy of data analysis (23). The Mixed Methods Appraisal Tool (MMAT) was selected because the included studies comprised qualitative, quantitative, and mixed methods designs. The MMAT allows consistent and transparent appraisal across heterogeneous methodologies within a single framework, facilitating comparability while maintaining methodological rigour. Although design-specific appraisal tools may provide more granular detail for individual study types, their combined use would have reduced coherence across the review. The MMAT was therefore considered the most appropriate tool given the objectives and mixed evidence base of this review.

All included studies were evaluated according to the relevant MMAT criteria and rated as “Yes,” “No,” or “Can't Tell” based on compliance with methodological standards. Quality assessment was conducted systematically to ensure methodological transparency and reliability. The MMAT checklist was applied independently to all included studies, and the results are presented in the Results section.

The purpose of the quality assessment was not to exclude studies based on methodological quality, but rather to support interpretation of the strength, credibility, and limitations of the synthesized evidence. This approach enabled a more informed evaluation of the findings and their implications for nursing practice and healthcare policy.

### 2.8 Data synthesis

Data were synthesized using a combination of narrative and thematic synthesis, which was considered the most appropriate approach given the heterogeneity of the 17 included studies in terms of research design, populations, settings, and reported outcomes (24). Data extraction was conducted by the primary reviewer, including study characteristics, methodology, and key findings related to barriers to cardiac rehabilitation participation. Thematic synthesis was initially undertaken by the primary reviewer through iterative coding and theme development across included studies. Emerging themes were reviewed, discussed, and refined collaboratively with the research team to ensure analytical rigor, consistency, and consensus. Any disagreements during data interpretation were resolved through discussion.

Narrative synthesis enabled the integration of qualitative, quantitative, and mixed-methods evidence into a coherent summary of barriers to cardiac rehabilitation participation among patients with heart failure and the role of nursing interventions in addressing these barriers. Thematic analysis was conducted according to the framework proposed by Braun and Clarke (2006) (25). This process involved familiarization with the extracted data, generation of initial codes, identification of recurring patterns, and classification of these patterns into broader themes.

Four overarching themes emerged from the analysis: (1) patient-related barriers, (2) systemic and structural barriers, (3) socio-cultural and equity barriers, and (4) nursing-related facilitators and support models. These themes provided a structured framework for synthesizing evidence and exploring the multidimensional factors affecting cardiac rehabilitation participation among patients with heart failure. This approach facilitated meaningful interpretation of the evidence and informed implications for nursing practice, healthcare service delivery, and policy development (26).

## 3 Results

### 3.1 Study selection

A comprehensive search of PubMed, CINAHL, EMBASE, Scopus, and MEDLINE identified 1,509 records. After removal of 146 duplicate records, 1,363 records remained for title and abstract screening. Following screening, 1,221 records were excluded. The remaining 142 articles underwent full-text assessment for eligibility. Of these, 125 studies were excluded for predefined reasons, including systematic reviews (*n* = 17), non-English publications (*n* = 10), studies involving cardiac conditions other than heart failure (*n* = 63), inappropriate populations (*n* = 25), and studies that did not examine barriers to cardiac rehabilitation (*n* = 10). A total of 17 studies met the inclusion criteria and were included in the final narrative and thematic synthesis. The study selection process is presented in Figure 1.

### 3.2 Characteristics of included studies

The final review included 17 studies published between 2019 and 2024. As summarized in Table 1, the studies were conducted across a range of countries, predominantly in high-income settings. The United States contributed the largest number of studies (*n* = 5) (27–31), followed by Australia (*n* = 2) (32, 33) and Denmark (*n* = 2) (34, 35). Additional studies were conducted in Scotland (36), South Korea (37), Portugal (38), Saudi Arabia (39), China (40), Germany/The Netherlands (41), and the United Kingdom/Ireland (42), while one study evaluated a digital cardiac rehabilitation programme without specifying a single-country setting.

**Table 1.** Studies which included characteristics.

No.	Study	Title	Study Design	Setting/Country	Sample Size	Aim of the Study	Main Outcome/Focus	Barriers Identified
1	Alonso et al. (27)	HEART Camp intervention study	RCT	USA	204 HF patients	To evaluate the HEART Camp intervention	Adherence, function, QoL	No separate barrier list was reported in the accessible abstract; the paper focused on improving already-poor exercise adherence in HFpEF through the HEART Camp intervention.
2	Kim et al. (37)	CR barriers comparison study	Cross sectional Quantitative survey	South Korea	Hospitals	To compare CR barriers	System-level barriers	Organisational and policy barriers: weak perceptions of CR effectiveness, negative views about patients' role in CR, weaker support in non-PCI/non-CR hospitals, and the need for timely referral, education, and government socioeconomic support
3	Al-Sharifi et al. (35)	CR access barriers among migrants	Qualitative	Denmark	Migrants	To explore barriers to CR access	Access and provision	Language-related barriers, including inadequate provision of CR core components for non-Danish-speaking patients and suboptimal interpreter services.
4	Daw et al. (42)	Home-based CR implementation barriers	Mixed methods/Qualitative semi structed interview -cross section online survey.	UK/Ireland	Healthcare staff	To identify implementation barriers	Implementation	Implementation barriers to REACH-HF within NHS services; the accessible abstract confirms service-implementation barriers but does not expose the full detailed barrier list.
5	Foster et al. (36)	Rural CR factors	Cross sectional Survey	Scotland	295 referrals	To examine rural attendance factors	Attendance	Rural access barriers: age and health issues, distance, longer travel time and costs, class starting times, and poor information/communication
6	Palmer et al. (33)	Engagement barriers in CHF programmes	Survey	Australia	165 CHF programmes	To identify engagement barriers	Attendance	Poor health literacy, including among medical/health professionals, weak flow through the health system, and limited flexibility in program delivery.
7	Braver et al. (43)	Digital CR perception study	Qualitative	Not stated	Patients and nurses	To explore digital CR perceptions	Experience	Digital connectivity problems, administrative complexity, and difficulty establishing trust without face-to-face care.
8	Yang et al. (40)	Home-based CR barriers	Qualitative	China	16 CHF patients	To identify home-based barriers	Adherence	Lack of knowledge, activity intolerance, exercise fear, role conflict, and lower exercise self-efficacy.
9	Alexandre et al. (38)	Enrolment barriers in HFrEF	retrospective Cohort	Portugal	214 HFrEF patients	To assess enrolment barriers	Clinical factors	No medical referral, concomitant medical problems, patient refusal, and geographical distance to the hospital.
10	Beleigoli et al. (32)	Clinical effectiveness of cardiac rehabilitation and barriers to completion in patients of low socioeconomic status in rural areas: A mixed-methods study	Mixed methods/ Explanatory sequential (QUAN → QUAL)	Australia	312 + staff	To examine socioeconomic barriers	Completion	The study is framed around low socioeconomic status and rurality as the main context for non-completion; the accessible abstract does not list all qualitative sub-barriers explicitly.
11	DeLara et al. (30)	CR vs. Internet Access Study	Cross-sectional	USA	3,142 counties	To assess CR and internet access	Access	Geographic access barriers, especially CR deserts; county-level inequities were also seen by race/ethnicity, education, and income. Broadband access was generally not the main limitation, which is why hybrid CR was suggested.
12	Chen et al. (29)	Geographic access and vulnerability	Ecological/Ecological Cross-sectional Study	USA	Medicare	To explore geographic access	Vulnerability	Worse geographic access to CR among socially vulnerable and rural communities.
13	Aldhahir et al. (39)	Nursing barriers in CR delivery	Cross-sectional	Saudi Arabia	412 nurses	To examine nursing barriers	Provider issues	From the nurse perspective, the main barriers were limited CR facility availability, treatment cost, and the absence of a standardised referral strategy.
14	Eichner et al. (31)	Virtual CR predictors	Observational Cohort	USA	185 patients	To identify predictors of virtual CR use	Attendance	Not a barrier-mapping study; it identified predictors of lower compliance in virtual CR: older age, diabetes, CVD diagnoses, and smoking at admission.
15	Aaby et al. (34)	Health literacy and participation	Cross-sectional	Denmark	1,435 patients	To examine health literacy	Participation	Health literacy was not associated with participation in CR; among nonrespondents, lower education and living alone were more common, but the study did not identify HL as the barrier itself.
16	Vonk et al. (41)	Nonattendance and dropout	Survey	Germany/Netherlands	850 patients	To identify reasons for nonattendance	Dropout	Main reasons for nonattendance/noncompletion were motivational and perceptual: not needing supervision, not needing the CR trajectory, already exercising regularly, other physical problems, not needing the trajectory, and the program not being personal enough.
17	Boggess et al. (28)	Nurse-led CR and dropout	Pre–post (before-and-after) study without a control group/ MIXED METODS	USA	500 + referrals	To assess nurse-led CR	Dropout	The study addressed delay between Phase 1 and Phase 2 enrollment; the intervention reduced the transition period, suggesting that late referral/readiness was the key problem.

Methodologically, the included studies comprised a variety of research designs, including one randomized controlled trial, two mixed-methods studies, three qualitative studies, and several quantitative observational, descriptive, and cross-sectional studies. Sample sizes varied considerably, ranging from 16 participants in qualitative investigations to population-level studies involving over 3,000 counties and national datasets. This methodological diversity enabled exploration of barriers to cardiac rehabilitation from patients, providers, organizational, and healthcare-system perspectives.

The included studies primarily examined patient-related barriers, systemic and structural barriers, socio-cultural determinants of participation, and nursing-related facilitators of cardiac rehabilitation engagement. Frequently reported barriers included inadequate referral pathways, geographic isolation, limited access to rehabilitation services, health literacy challenges, exercise-related fear, socioeconomic disadvantages, and language barriers. Several studies also examined nurse-led interventions, care coordination strategies, and digital or home-based rehabilitation models designed to improve enrolment, adherence, and programme completion. Detailed characteristics of the included studies are presented in Table 1.

### 3.3 Quality assessment

The methodological quality of the 17 included studies was assessed using the Mixed Methods Appraisal Tool (MMAT) 2018 (22). Overall methodological quality varied across study designs. Experimental and cohort studies generally demonstrated clearer eligibility criteria and outcome measurement, strengthening internal validity. In contrast, cross-sectional surveys frequently lacked justification for sample representativeness and were more susceptible to non-response bias. Qualitative studies consistently demonstrated coherence between research questions, data collection, and analytical approaches; however, limited reflexivity and insufficient reporting of researcher positionality were common. Across all study types, limited adjustment for confounding factors constrained causal interpretation. These methodological limitations should be considered when interpreting the findings, although thematic consistency across studies supports the credibility of identified barriers. Detailed quality assessment findings are presented in Table 2. Taken together, although methodological limitations (particularly the predominance of observational designs and limited control for bias) restrict causal inference, the consistency of findings across diverse study designs supports a moderate to high level of confidence in the review's main conclusions regarding barriers to cardiac rehabilitation participation.

**Table 2:** MMAT quality appraisal table.

Study (Author, Year)	Study Design	S1	S2	C1	C2	C3	C4	C5	Quality Score	Comments
Alonso et al., 2022 (27)	RCT	Y	Y	Y	Y	Y	Y	Y	High	Well-designed RCT; clear outcomes; some performance bias possible
Kim et al., 2022 (37)	Quantitative survey	Y	Y	Y	Y	Y	N/A	N/A	Moderate	Clear research question; representative sample; some reporting bias possible
Al-Sharifi et al., 2019 (35)	Qualitative	Y	Y	Y	Y	Y	N/A	N/A	High	Rich qualitative data; clear methodology; appropriate interpretation
Daw et al., 2022 (42)	Mixed methods	Y	Y	Y	Y	Y	Y	Y	High	Robust mixed methods; clear integration of qualitative and quantitative data
Foster et al., 2021 (36)	Quantitative survey	Y	Y	Y	Y	Y	N/A	N/A	Moderate-High	Good sample size; clear methodology; some nonresponse bias possible
Palmer et al., 2020 (33)	Quantitative survey	Y	Y	Y	Y	CT	N/A	N/A	Moderate	Service-based survey; useful CHF-specific data; response bias possible
Braver et al., 2023	Qualitative	Y	Y	Y	Y	Y	N/A	N/A	High	Strong qualitative methodology; dual perspective (patients and nurses)
Yang et al., 2023 (40)	Qualitative Descriptive	Y	Y	Y	Y	Y	N/A	N/A	High	Clear descriptive methodology; rich patient perspectives
Alexandre et al., 2022 (38)	Observational cohort	Y	Y	Y	Y	Y	Y	N/A	High	Well-conducted retrospective cohort; clear clinical determinants
Beleigoli et al., 2024 (32)	Mixed methods	Y	Y	Y	Y	Y	Y	Y	High	Excellent integration; addresses vulnerable populations
DeLara et al., 2024 (39)	Cross-sectional	Y	Y	Y	Y	Y	N/A	N/A	High	Large sample (all US counties); innovative focus on digital infrastructure
Chen et al., 2024 (29)	Ecological cross-sectional	Y	Y	Y	Y	Y	N/A	N/A	Moderate-High	Nationwide Medicare data; clear geographic analysis
Aldhahir et al., 2022 (39)	Cross-sectional	Y	Y	Y	Y	Y	N/A	N/A	High	Large nursing sample (*n* = 412); national survey; directly addresses nursing perspectives
Eichner et al., 2023 (31)	Observational cohort	Y	Y	Y	Y	CT	Y	N/A	Moderate	Virtual CR focus; clear predictors; some attrition concerns
Aaby et al., 2020 (34)	Cross-sectional	Y	Y	Y	Y	Y	N/A	N/A	High	Very large sample (*n* = 1,435); robust health literacy measurement
Vonk et al., 2021 (41)	Cross-sectional survey	Y	Y	Y	Y	Y	N/A	N/A	High	Large sample (*n* = 850); clear patient-reported reasons for nonattendance
Boggess et al., 2024 (28)	Retrospective pre-/post-intervention	Y	Y	Y	Y	CT	Y	N	Moderate	Nursing intervention focus; large referral sample; retrospective limits

S1, Clear research question? S2, Data address research question? C1-C5, Design-specific criteria Y, Yes, N, No, CT, Can't tell, N/A, Not applicable.

### 3.4 Thematic synthesis

Data from the 17 included studies were synthesized using a combination of narrative and thematic synthesis. This approach was considered appropriate because of the heterogeneity of the included studies with respect to study design, populations, settings, and reported outcomes. The synthesis enabled the integration of qualitative, quantitative, and mixed-methods evidence to identify barriers to cardiac rehabilitation participation among patients with heart failure and the role of nursing in addressing these barriers.

Thematic analysis was conducted according to the framework proposed by Braun and Clarke (2006) (25). Following familiarization with the extracted data, initial codes were generated and subsequently grouped into broader categories reflecting common patterns across studies. Four overarching themes emerged from the analysis: (1) patient-related barriers, (2) systemic and structural barriers, (3) socio-cultural and equity barriers, and (4) nursing-related facilitators and support models. The thematic synthesis matrix is presented in Table 3.

**Table 3:** Thematic synthesis matrix of included studies.

Main theme	Subthemes	Supporting studies
1. Patient-related barriers	- Clinical and demographic factors (age, comorbidities, disease severity, ejection fraction eligibility) - Psychological and motivational barriers (low motivation, fear, perceived burden, low confidence) - Physical symptoms and home-based adherence challenges (fatigue, pain, functional limitation) - Health literacy and patient understanding - Reasons for nonattendance and noncompletion reported by patients	- Alexandre et al. (38) - Alonso et al. (27) - Yang et al. (40) - Aaby et al. (34) - Vonk et al. (41)| - Palmer et al. (33)
2. Systemic and structural barriers	- Limited availability of CR services and institutional capacity - Weak referral pathways and implementation barriers - Geographic and rural access barriers - Low socioeconomic status and service inequity - Infrastructure barriers affecting access to care	- Kim et al. (37) - Daw et al. (42) - Foster et al. (36) - Palmer et al. (33) - Beleigoli et al. (32) - Chen et al. (29) - DeLara et al. (30) - Aldhahir et al. (39)
3. Socio-cultural and equity barriers	- Language barriers affecting access to CR - Migrant and equity-related barriers - Socioeconomic disadvantage - Broader adherence barriers in under-served groups	- Al-Sharifi et al. (35) - Beleigoli et al. (32) - Foster et al. (36)
4. Nursing-related facilitators and models of support	- Nursing perspectives on barriers to CR delivery - Patient education and motivational support - Nurse-led care coordination - Digital and tele-rehabilitation models - Predictors of attendance and compliance in virtual CR	- Aldhahir et al. (39) - Braver et al. (43) - Boggess et al. (28) - Daw et al. (42) - Eichner et al. (31)

### 3.5 Thematic synthesis of findings

#### 3.5.1 Theme 1: patient-related barriers

Patient-related barriers significantly hinder engagement in and completion of recommended cardiac rehabilitation. Within this theme, the main empirical literature underscores a complex interaction of demographic, clinical, psychological, and cognitive elements that distinctly define the heart failure population.

##### 3.5.1.1 Clinical complexity, aging, and symptom intolerance

The clinical symptoms of heart failure significantly hinder conventional rehabilitation routes. Older age and significant multi-morbidity serve as substantial structural baseline barriers that directly reduce enrollment speed. Alexandre et al. (38) investigated barriers to enrollment in a specific cohort with Heart Failure with Reduced Ejection Fraction (HFrEF), revealing that increased clinical complexity and acute accompanying medical issues often hinder patient inclusion or result in direct exclusion by physicians. Moreover, even when patients are successfully enrolled, their daily compliance is significantly affected by ongoing physical symptoms. Significant limitations in functional activity, fluid accumulation, and ongoing tiredness render it exceptionally difficult for patients with chronic heart failure to stick to home-based exercise routines (34, 40).

##### 3.5.1.2 Psychological resistance and fear-avoidance behaviours

Aside from physical constraints, mental obstacles significantly influence absenteeism and early withdrawals. Patients with heart failure often exhibit significant “fear-avoidance” behaviors, limiting their movement due to the association of physical activity with the distressing experience of severe dyspnea (40). This anxiety is heightened by low baseline self-confidence and a significant perceived treatment burden. Vonk et al. (41) investigated self-reported causes of non-attendance in a sizable sample of 850 patients, finding that dropout rates are primarily influenced by cognitive misunderstandings; numerous patients indicated that professional oversight was unnecessary or thought their individual daily tasks were adequate. Alonso et al. (27) emphasized that behavioral interventions should directly address these low exercise-adherence attitudes in heart failure patients to avoid psychological disengagement.

##### 3.5.1.3 Health literacy deficits and digital readiness

Cognitive and social traits also influence a patient's ability to manage rehabilitation. Limited health literacy acts as a significant obstacle; when patients or their close family units fail to grasp the physiological benefits of secondary prevention, adherence to long-term programs declines drastically (33). Aaby et al. (34) interestingly found that although low formal education and living alone were common among non-respondents, health literacy scores by themselves did not determine structural participation rates, indicating that social isolation may have a more significant impact. Additionally, with healthcare systems shifting to hybrid models, digital preparedness has surfaced as a new obstacle for patients. Braver et al. (43) and Eichner et al. (31) showed that engagement in virtual telerehabilitation relies not only on the availability of technology but also on patients' perceptions, confidence, and their ability to adapt to digital tracking systems.

#### 3.5.2 Theme 2: systemic and structural barriers

The analysis reveals that, at the institutional level, the effective implementation of cardiac rehabilitation is significantly hindered by inflexible organizational boundaries, disjointed referral systems, and critical resource shortages. These systemic issues merge to produce significant disparities in access to health services.

##### 3.5.2.1 Fragmented referral pathologies and institutional capacity barriers

The studies highlight a significant gap in the shift from acute hospital stabilization to rehabilitation in the community. Ineffective, manual medical referral systems constitute a major institutional shortcoming that results in many qualified heart failure patients not being referred (37, 38). In a nationwide assessment of South Korean hospitals, Kim et al. (37) found that organizational obstacles—like poor administrative views on CR effectiveness and limited support in non-percutaneous coronary intervention (non-PCI) facilities—considerably hinder care transitions. This institutional failure is reflected by the healthcare providers as well; Daw et al. (42) pointed out that healthcare personnel striving to execute structured programs (like the REACH-HF protocol within the NHS) encounter significant barriers to implementation, such as extreme limitations on staff time and a total absence of committed institutional funding.

##### 3.5.2.2 Geographic isolation

Physical distance and geographic isolation serve as an overwhelming obstacle for many individuals with heart failure. Patients in rural areas experience significant geographical obstacles, dealing with long travel distances, extended travel durations, and steep out-of-pocket expenses for transportation to access centralized healthcare facilities (36, 38). Chen et al. (29) conducted a comprehensive ecological cross-sectional study utilizing nationwide Medicare data, demonstrating that geographic access to rehabilitation centers is consistently poorer for communities marked by high social vulnerability and rural characteristics. This geographic separation establishes a structural “postcode lottery,” in which a patient's location wholly determines their access to care. DeLara et al. (30) assessed this infrastructure deficiency across 3,142 US counties, verifying the prevalent existence of “cardiac rehabilitation deserts” counties entirely lacking local rehabilitation facilities.

##### 3.5.2.3 Infrastructure gaps in digital delivery

To address these physical access obstacles, health systems have progressively sought to implement virtual telerehabilitation approaches. Nonetheless, the organized application of these models is limited by significant infrastructure shortcomings. DeLara et al. (30) pointed out that although the availability of broadband internet wasn't the sole limiting factor for every group, significant county-level disparities due to income and education greatly restrict the equity of hybrid delivery. Aldhahir et al. (39) conducted a survey of 412 nurses, revealing that the absence of standardized telerehabilitation systems, along with elevated treatment expenses and restricted institutional capacity, hinders healthcare professionals from efficiently implementing remote monitoring processes. This structural shortcoming indicates that instead of bridging the access gap, digital health models may exacerbate a digital divide for heart failure populations with limited resources.

#### 3.5.3 Theme 3: socio-cultural and equity barriers

The thematic synthesis indicates that traditional, center-focused cardiac rehabilitation models often do not meet the needs of varied and structurally marginalized patient groups. Socio-cultural differences, language barriers, and profound economic hardship combine to present substantial compliance challenges, leading to markedly reduced enrollment and elevated dropout rates in programs for minority and low-income heart failure populations.

##### 3.5.3.1 Linguistic isolation and cultural marginalization

Linguistic differences between healthcare delivery systems and local communities create significant communication obstacles that essentially prevent ethnic minority groups from engaging in cardiac rehabilitation. Al-Sharifi et al. (35) examined the qualitative access experiences of migrant communities, showing that language obstacles lead to consistent care dropouts. When essential educational elements, lifestyle guidance sessions, and physical training directives are provided solely in a prevailing national language, patients who are non-native speakers face significant psychological isolation. This exclusion is greatly worsened by disjointed, inadequate clinical interpreter services that do not effectively communicate intricate medical guidance during extended exercise periods, resulting in language-minority patients lacking the crucial safety knowledge needed to maintain program participation.

##### 3.5.3.2 Financial difficulty and the cycle of socioeconomic deprivation

Personal financial difficulties directly affect access to healthcare systems, resulting in a cycle of low enrollment and elevated non-completion rates among patients from socioeconomically disadvantaged backgrounds. Patients with heart failure who come from low-income and low-education backgrounds face urgent, tangible financial obstacles that render adherence to center-based programs unfeasible. According to Beleigoli et al. (32), low socioeconomic status serves as a primary factor for non-completion, influenced by an absence of private transportation, the inability to cover out-of-pocket program expenses, and insufficient social support networks. Moreover, these financial limitations are intensified by inflexible facility arrangements; low-income patients frequently cannot afford to miss work or obtain adaptable transportation to align with set daytime clinical rehabilitation schedules (36). As a result, traditional delivery models unintentionally perpetuate healthcare disparities by not addressing the everyday survival needs of economically disadvantaged communities.

#### 3.5.4 Theme 4: Nursing-Related Facilitators and Support Models

A key discovery in the compiled literature is that nursing professionals serve as the most essential link between the stabilization of acute heart failure and ongoing community rehabilitation. With the implementation of structured clinical care pathways, frontline nurses effectively utilize their positions as coordinators, educators, and technology guides to systematically address the patient-level and organizational obstacles highlighted in earlier themes.

##### 3.5.4.1 Structural coordination and transitional care management

Nurses act as the main clinical designers of discharge transitions, directly addressing institutional referral failures and reducing administrative lapses. Conventional care delivery frequently experiences extended transactional delays that result in eligible heart failure patients being overlooked during their transition from inpatient wards to community services. Boggess et al. (28) assessed a systematic nurse care coordination initiative, demonstrating that proactive, nurse-led monitoring effectively closes this care transition gap. Through the use of specialized workflows to detect suitable patients promptly at the bedside and implementing effective referral management, nurse coordinators significantly enhance enrollment speed and minimize the risk of patients exiting the care continuum during critical post-discharge periods.

##### 3.5.4.2 Behavioural, counselling, educational advocacy, and trust

In addition to handling institutional logistics, nursing staff are specifically equipped to identify and address the cognitive, emotional, and motivational obstacles faced by heart failure patients. Symptom fluctuation often leads patients to experience significant fear-avoidance regarding exercise and diminished self-efficacy. Frontline nurses work to break down these psychological obstacles by providing personalized health education, specific patient counseling, and motivational interviewing before hospital discharge (42). By customizing clinical safety communication to match a patient's specific health literacy level, nurses effectively transform the perception of physical activity from a potential danger into a secure, empowering treatment resource. This focused relational assistance directly tackles the significant motivation declines identified in patient surveys, turning inactive non-attendees into engaged participants.

##### 3.5.4.3 Clinical anchors and digital navigation in telerehabilitation

As healthcare systems progressively transition to virtual and hybrid delivery models to address geographic obstacles, the clinical scope of nursing has swiftly grown to include remote digital monitoring. Although virtual platforms may alleviate travel challenges, they also create significant technical issues, administrative difficulties, and obstacles in electronic communication for older heart failure patients. Braver and colleagues (2023) showed that telerehabilitation nurses act as essential technical and clinical support within these digital environments. Nurses effectively handle administrative intricacies, assist patients with digital platforms, resolve connectivity issues, and oversee clinical safety monitors from a distance. Importantly, ongoing nursing engagement creates profound relational trust in virtual settings, successfully compensating for the lack of in-person interaction and directly supporting long-term adherence to exercise and data monitoring in digital contexts (31, 39).

### 3.6 Facilitators and strategies to improve participation

Despite the widespread existence of multi-level obstacles, the reviewed literature recognized several effective clinical enablers and evidence-based approaches that can significantly improve cardiac rehabilitation (CR) engagement among heart failure (HF) patients. The randomized controlled trial (RCT) by Alonso et al. (27) shows that structured, behaviorally supported intervention models (like the HEART Camp protocol) result in notable improvements in long-term exercise adherence, functional capacity measures, and health-related quality of life. This directly suggests that shifting from passive care models to well-structured, goal-focused rehabilitation pathways produces measurable clinical benefits for heart failure groups.

Moreover, the main literature emphasizes that the structural effectiveness of these programs improves when interventions are actively led by nursing staff. Boggess et al. (28) illustrated the essential role of nurse-led care coordination in reducing program attrition, showing that thorough nursing follow-up, ongoing clinical monitoring, and automated discharge processes significantly enhance long-term patient retention. To address the physical and geographic access challenges outlined earlier, the literature strongly supports the implementation of decentralized digital health frameworks, if they are organized according to patient capabilities. Eichner et al. (31) demonstrated that virtual telerehabilitation serves as a very effective substitute for center-based care, highlighting that better outcomes arise when healthcare providers actively monitor and address clinical predictors of reduced compliance, like older age or metabolic comorbidities. Likewise, Daw et al. (42) and Braver et al. (43) proposed that home-based, online, and hybrid delivery models effectively navigate the significant structural issues of geographic isolation, travel distances, and transportation expenses

In conclusion, the key facilitators highlighted in the primary evidence are those that enhance ongoing clinical assistance while reducing the practical and logistical challenges faced by the patient. These ideal frameworks include nurse-led transitional routes, home-centered virtual systems, and rigorously organized behavioral strategies that involve ongoing monitoring. The evidence shows that heart failure rehabilitation outcomes will greatly enhance when services are provided through accessible, adaptable, and well-coordinated processes.

## 4 Discussion

This systematic review explored barriers to cardiac rehabilitation (CR) participation among patients with heart failure (HF) and examined the role of nursing in improving referral, enrolment, participation, and adherence. The findings demonstrate that CR participation is influenced by a complex interaction of patient, organizational, health-system, and socio-cultural factors rather than being solely dependent on patient motivation.

### 4.1 Principal findings

The screening process revealed that from 1,509 initially identified records, only 17 studies met the inclusion criteria, with exclusion most commonly due to studies focusing on cardiac conditions other than heart failure. This pattern highlights the relative scarcity of evidence specifically addressing cardiac rehabilitation participation within heart failure populations. Much of the existing evidence remains embedded within broader cardiac rehabilitation literature, limiting the specificity of recommendations for heart failure care. This gap underscores the need for more targeted research examining rehabilitation access, delivery, and nursing-led interventions tailored to heart failure populations.

The findings of this review demonstrate that barriers to cardiac rehabilitation participation occur at multiple levels. At the patient level, barriers included health literacy limitations, exercise-related fear, digital readiness challenges, and the clinical complexity of heart failure. At the provider and organizational level, fragmented referral pathways, limited staffing, and restricted service availability were frequently identified. At the system level, geographic inequities, socioeconomic disadvantages, and digital access limitations further reduce participation in rehabilitation programs. Conversely, nurse-led care coordination, home-based and hybrid rehabilitation models, virtual rehabilitation supported by nursing follow-up, and structured behavioral interventions were identified as important facilitators of enrolment and adherence.

### 4.2 Comparison with literature

The multi-level barriers noted in this review reflect wider patterns in the literature on chronic cardiovascular care. The recognized patient factors like age, multi-morbidity, and financial stress correspond with Sugiharto et al. (44) concerning coronary heart disease groups, and Iyngkaran et al. (45) concerning low and middle-income nations (LMICs). This review highlights that patients with heart failure experience a distinctively unstable symptom progression, which exacerbates these typical obstacles.

#### 4.2.1 Health literacy and referral pathways

The findings of this review align with those reported by Beauchamp et al. (46), who observed that although low health literacy may not always predict final attendance, it can substantially limit patients' understanding of referral pathways and rehabilitation opportunities, who noted that, although low health literacy may not always predict final attendance, it significantly limits a patient's early understanding of referral options. This review expands on Isselhard et al. (47) by claiming that health literacy is a flexible resource instead of an unchanging barrier. By adjusting CR programs to accommodate specific literacy levels with customized nursing explanations and guidance, healthcare systems can effectively enhance patient self-efficacy.

#### 4.2.2 Socio-cultural responsiveness

The socio-cultural obstacles identified in this review (35) are strongly supported by Vanzella et al. (48) and Tofani et al., who contend that ongoing systemic inequalities arise because conventional CR frameworks do not deliver linguistically responsive care or sufficient interpreter incorporation. In the same vein, Vanzella et al. 24 observe that family expectations and cultural values significantly impact minority communities. This supports your analysis: conventional, inflexible center-based CR models intended for majority groups lack the clinical adaptability needed to cater to varied cultural and linguistic backgrounds (49).

#### 4.2.3 Spatial inequities

Geographically, recognizing “CR deserts” and significant regional inequalities directly relates to Mathews and Brewer (50) and Duncan et al. (11), who noted that state-level CR initiation varies dramatically from 10% to 40%. Chen et al. (29) further validates this structural deficit, confirming that communities with social vulnerabilities endure considerably longer travel distances to access physical facilities. Although the digital shift helps mitigate this spatial disparity, Gallegos-Rejas et al. (51) warn that the effective, successful aspects of telehealth CR have not yet been distinctly separated from those that are unsuccessful.

As emphasized by Brown et al. (52) and Taylor et al. (10), effective multidisciplinary care coordination, thorough patient evaluation, and ongoing behavioral counseling are key factors in enhancing quality of life and functional capacity. In this framework, nurses are not an auxiliary component; they are the main forces propelling patient recruitment, education, and sustained retention.

### 4.3 Implications for nursing practice

The results suggested that nurses should actively seek to identify obstacles early in the referral and discharge procedures. These encompass knowledge of health, travel and transportation challenges, digital accessibility, language proficiency, and self-management abilities. Nurses can also improve adherence through teaching, motivational interviewing, cultural sensitivity, and following up with patients who discontinue or face attendance issues (52). In this context, Taylor et al. (10) also proposed that these programs are most effectively provided as an active service rather than a passive one. Therefore, nursing support must persist regardless of whether the delivery occurs online or at the patient's residence. In nursing education, CR must be incorporated into cardiovascular secondary prevention instead of functioning as a specialized service. Cardiovascular risk reduction must be included in pre-registration and ongoing professional development, emphasizing the principles of CR referral, exercise rehabilitation, health literacy, motivational interviewing, telehealth, and skills for effective cultural communication. CR's fundamental aspects are multidisciplinary and centered on the patient, which means nursing students and professionals need to recognize CR's advantages and the practice elements that adversely affect patient participation (53). Consequently, nurses need training to provide face-to-face, hybrid, and virtual CR. The review recommends automatic referrals, enhanced service coverage, and adaptable service delivery for policy and service organizations. Healthcare systems ought to emphasize hybrid CR, transportation, internet access, and staffing to guarantee that access remains unaffected by location, financial status, or digital skills. Policy must emphasize culturally and linguistically appropriate services for migrant and minority populations, in addition to outreach programs for rural and socially disadvantaged communities.

### 4.4 Strengths and limitations of the review

Several limitations should be acknowledged. First, the review was restricted to English-language publications, which may have resulted in the exclusion of relevant studies from non-English-speaking regions and introduced language bias. Second, most included studies originated from high-income countries, particularly the United States, Australia, and Denmark, which may limit the generalizability of the findings to low- and middle-income settings where cardiac rehabilitation infrastructure and nursing roles may differ. Third, the evidence base was predominantly observational, with only one randomized controlled trial included, thereby constraining the ability to draw causal inferences regarding the effectiveness of nurse-led interventions. These limitations should be considered when interpreting the findings.

### 4.5 Future research

Future research should evaluate the effectiveness of nurse-led, hybrid, and digitally enabled cardiac rehabilitation models across diverse healthcare settings. Particular attention should be given to underserved populations, including women, migrants, rural communities, and patients with low health literacy. Further studies are also required to explore referral pathways, digital exclusion, and long-term adherence strategies to support equitable access to cardiac rehabilitation services.

## 5 Conclusion

This systematic review demonstrated that barriers to cardiac rehabilitation (CR) participation among patients with heart failure (HF) are complex and multidimensional. Barriers were identified at the patient, provider, organizational, and health-system levels, including clinical complexity, health literacy limitations, exercise-related fear, fragmented referral pathways, geographic isolation, digital accessibility challenges, and socioeconomic disadvantages. The findings also highlight persistent inequities affecting women, migrants, rural populations, and individuals from lower socioeconomic backgrounds.

The review further identified nursing as a critical facilitator of successful CR participation. Nurse-led education, care coordination, motivational support, referral management, and ongoing follow-up were consistently associated with improved enrolment, adherence, and programme completion. Although home-based, digital, and hybrid CR models offer promising solutions to overcome geographic and logistical barriers, their effectiveness depends on continued clinical supervision and patient-centered support.

Overall, the findings suggest that CR participation should be viewed as an issue of implementation and healthcare equity rather than a matter of patient motivation alone. Improving participation in CR requires coordinated action across clinical practice, service delivery, workforce development, and health policy. Strengthening nurse-led interventions and expanding flexible, equitable rehabilitation models may improve cardiac rehabilitation referral, engagement, and adherence among people living with heart failure.
